# Cloning and expression of *BpMYC4* and *BpbHLH9* genes and the role of *BpbHLH9* in triterpenoid synthesis in birch

**DOI:** 10.1186/s12870-017-1150-z

**Published:** 2017-11-21

**Authors:** Jing Yin, Xin Li, Yaguang Zhan, Ying Li, Ziyue Qu, Lu Sun, Siyao Wang, Jie Yang, Jialei Xiao

**Affiliations:** 1 College of Life Science, Northeast Forestry University, Harbin, 150040 China; 2 State Key Laboratory of Tree Genetic Breeding, Northeast Forestry University, Harbin, 150040 China; 3 College of Agriculture, Northeast Agriculture University, Harbin, 150010 China

**Keywords:** *Betula platyphylla*, *BpMYC4*, *BpbHLH9*, Expression characteristics, Triterpenoids

## Abstract

**Background:**

Birch (*Betula platyphylla* Suk.) contains triterpenoids with anti-HIV and anti-tumor pharmacological activities. However, the natural abundance of these triterpenoids is low, and their chemical synthesis is costly. Transcription factors have the ability to regulate the metabolite pathways of triterpenoids via multi-gene control, thereby improving metabolite yield. Thus, transcription factors have the potential to facilitate the production of birch triterpenoids. Plant bHLH (basic helix-loop-helix) transcription factors play important roles in stress response and secondary metabolism.

**Results:**

In this study, we cloned two genes, *BpMYC4* and *BpbHLH9*, that encode bHLH transcription factors in *Betula platyphylla* Suk. The open reading frame (ORF) of *BpMYC4* was 1452 bp and encoded 483 amino acids, while the ORF of *BpbHLH9* was 1140 bp and encoded 379 amino acids. The proteins of *BpMYC4* and *BpbHLH9* were localized in the cell membrane and nucleus. The tissue-specific expression patterns revealed that *BpMYC4* expression in leaves was similar to that in the stem and higher than in the roots. The expression of *BpbHLH9* was higher in the leaves than in the root and stem. The expressions of *BpMYC4* and *BpbHLH9* increased after treatment with abscisic acid, methyl jasmonate, and gibberellin and decreased after treatment with ethephon. The promoters of *BpMYC4* and *BpbHLH9* were isolated using a genome walking approach, and 900-bp and 1064-bp promoter sequences were obtained for *BpMYC4* and *BpbHLH9*, respectively. The ORF of *BpbHLH9* was ligated into yeast expression plasmid pYES3 and introduced into INVScl and INVScl1-pYES2-SS yeast strains. The squalene and total triterpenoid contents in the different INVScl1 transformants decreased in the following order INVScl1-pYES-SS-bHLH9 > INVScl1-pYES3-bHLH9 > INVScl1-pYES2- BpSS > INVScl-pYES2. In BpbHLH9 transgenic birch, the relative expression of the genes that encodes for enzymes critical for triterpenoid synthesis showed a different level of up-regulation compair with wild birch(control), and the contents of betulinic acid, oleanolic acid and betulin in bHLH9–8 transgenic birch were increased by 11.35%, 88.34% and 23.02% compared to in wild birch, respectively.

**Conclusions:**

Our results showed that the modulation of *BpbHLH9* by different hormones affected triterpenoid synthesis and triterpenoid contents. This is the first report of the cloning of *BpbHLH9*, and the findings are important for understanding the regulatory role of *BpbHLH9* in the synthesis of birch triterpenoids.

**Electronic supplementary material:**

The online version of this article (10.1186/s12870-017-1150-z) contains supplementary material, which is available to authorized users.

## Background

Birch contains important secondary metabolites, including betulin, betulinic acid, and oleanolic acid. Betulinic and oleanolic acids show potential for use in anticancer and anti-HIV therapeutics [1]. However, the development of natural medicines is limited by the scarcity of natural drugs, and the effective use of natural plant resources is critical. Because of the low contents of plant secondary metabolites and their limited distribution in particular species, organs, tissues, and cells, improving the yields of plant secondary metabolites is important for the development of natural drugs [2].

Terpene biosynthesis is regulated by enzyme genes and transcription factors. Transcription factors regulate biosynthesis by changing the level of gene expression through alterations in transcription rate. Therefore, it is critical to study the transcription factors related to terpene biosynthesis [3]. The Basic helix-loop-helix (bHLH) transcription factors have been shown to be involved in plant growth and developmental processes (e.g., the formation of trichome, photomorphogenesis, and light signal transduction) along with stress response and secondary metabolism [4], and a few bHLH transcription factors have been identified that modulate the biosynthesis of plant terpenes [5].

MYC2 is a bHLH transcription factor known to play a primary role in the jasmonate (JA) signaling cascade [6]. The homologues of MYC2 played important roles in regulating the genes involved in sesquiterpene biosynthesis in *Arabidopsis thaliana*, *Solanum lycopersicum*, *Catharanthus roseus*, and *Artemisia annua* [7–10]. In *Arabidopsis*, MYC2 protein interacted with DELLA proteins to upregulate the expression of sesquiterpene synthase genes in flowers by integrating the gibberellin (GA) and JA signaling pathways [8]. In the presence of TTG1 (a WD40 repeat-containing protein) along with MYC2 protein formed a transcriptional regulatory complex with MYB protein, thereby activating the genes involved in anthocyanin biosynthesis [11]. In *Catharanthus roseus*, CrMYC2 was associated with the expression of several genes related to the biosynthesis of terpenoid indole alkaloid (TIA), and CrMYC2 bound with the cis-elements of the *ORCA*3 gene promoter to induce TIA accumulation [7]. Similarly, the NbMYC2a/b proteins from tobacco (*N. tabacum*) upregulated the ORCA-related NIC2 locus to enhance nicotine biosynthesis [12]. Recently, Bl (bitter leaf) and Bt (bitter fruit) were reported to regulate cucurbitacin triterpene accumulation in *Cucumis sativus* [13]. In *Catharanthus roseus*, BIS1 was reported to control the monoterpene (iridoid) branch of the monoterpene indole alkaloid pathway [14]. These transcription factors belong to the bHLH family. Furthermore, two bHLH transcription factors (NbbHLH1 and NbbHLH2) were shown to positively regulate the accumulation of nicotine through binding to the G-box elements of the putrescine N-methyltransferase promoter; in contrast, BbbHLH3 was found to be a negative regulator [15].

Although bHLH transcription factors have been characterized in *Arabidopsis thaliana*, *Solanum lycopersicum* (tomato), *Catharanthus roseus*, *Artemisia annua*, and other plants, none have been characterized in *Betula platyphylla*. Triterpenoids form from 2,3-oxidized squalene, a precursor of the mevalonate pathway. As important nodes in the metabolic pathways of triterpenoids, the key enzymes 3-hydroxy-3-methyl glutaryl coenzyme A reductase (HMGR), farnesyl pyrophosphonate synthase (FPS), squalene synthase (SS), and squalene epoxidase (SE) were found to be directly involved in the synthesis of 2,3-oxidized squalene, and their contents and activities played important roles in regulating the synthesis and accumulation of terpenes [16, 17]. We previously showed that the promoters of HMGR, FPS, SS, and SE in *B. platyphylla* contained G-box elements regulated by bHLH transcription factors, and these bHLH transcription factors may influence the synthesis and accumulation of triterpenoids [2]. Our past work also suggested that *SS* and *SE* in *B. platyphylla* transformed the INVScl1 strain of yeast, as indicated by the significant increase in squalene content caused by the INVScl1 transformants [2]. Based on these previous results, we selected two transcription factors, MYC4 and bHLH9, from our laboratory transcriptome database. These transcription factors were demonstrated to respond to methyl jasmonate (MeJA) signaling and may play a role in the synthesis of birch triterpenes. We then cloned the full lengths of the genes encoding MYC4 and bHLH9 (*BpMYC4* and *BpbHLH9*, respectively) and analyzed their tissue-specific expression patterns after treatment with different phytohormones. In addition, we transferred *BpbHLH9* to the INVScl1-pYES2-BpSS yeast strain in an attempt to gain a greater understanding of the regulatory roles of *BpMYC4* and *BpbHLH9* in birch triterpenoid biosynthesis.

## Results

### Cloning and analysis of *BpMYC4* and *BpbHLH9*

Special primers were designed based on the nucleotide sequence in the transcriptome database of *B. platyphylla* (Additional file 1: Table S1). The full lengths of *BpMYC4* and *BpbHLH9* from *B. platyphylla* were isolated by real-time RT-PCR. The *BpMYC4* cDNA sequence length was 1564 bp and contained a 1452-bp open reading frame (ORF) encoding 483 amino acids (Additional file 2: Figure S1). Similarly, a *BpbHLH9* cDNA sequence of 1243 bp was obtained with a 1140-bp ORF encoding a protein of 379 amino acids (Additional file 3: Figure S2).

**Fig. 1:**
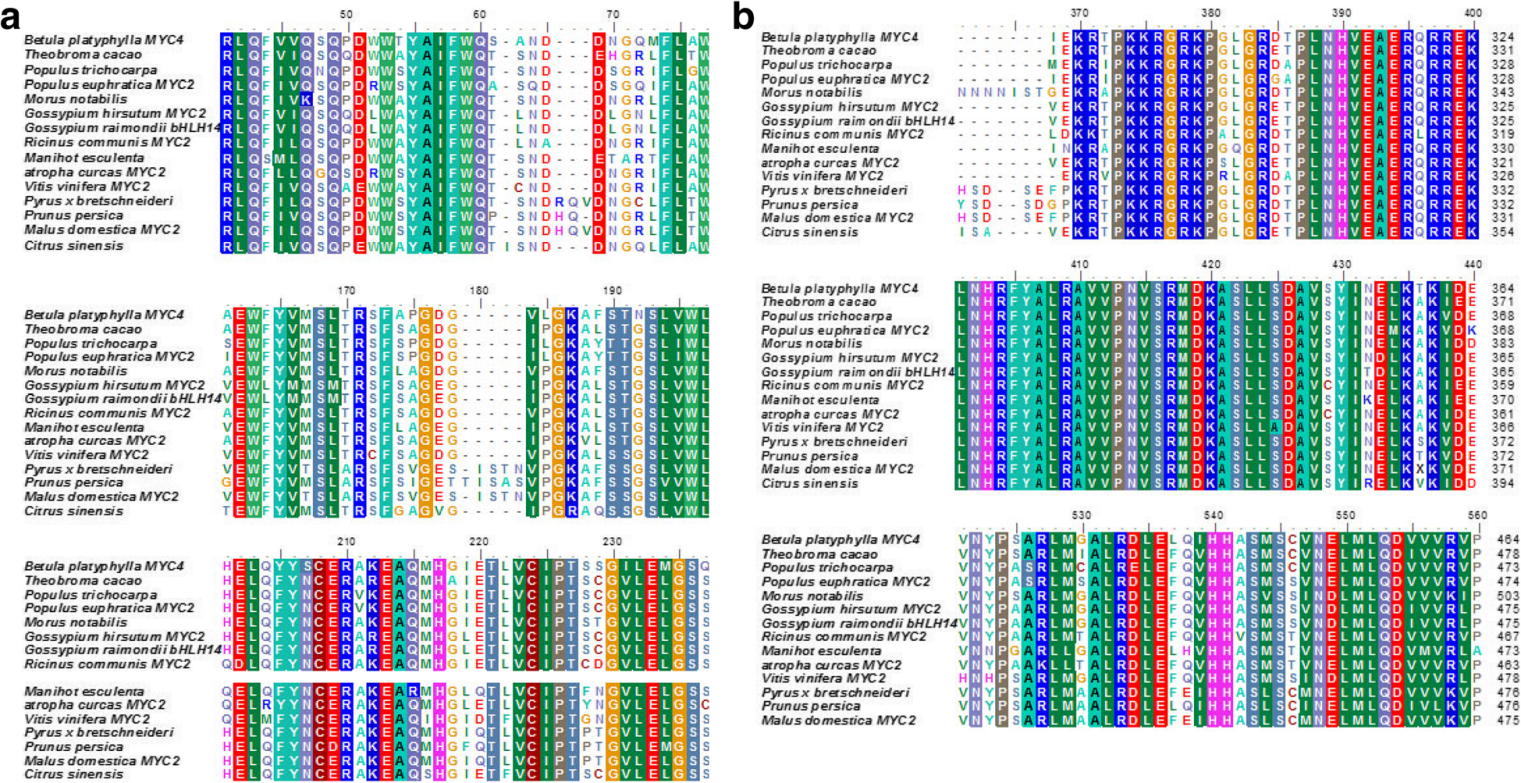
Alignment of the amino acid sequence encoded by *BpMYC4* with those encoded by the *bHLH*s of other organisms obtained using the ClustalW multiple alignment tool (**a**, **b**). The aligned sequences were derived from *Theobroma caca* (XP_007051457.1), *Populus trichocarpa* (XP_002301432.1), *Populus euphratica MYC2* (XP_011039024. 1), *Morus notabilis* (XP_010100678.1), *Gossypium hirsutum MYC2* (XP_016695799.1), *Gossypium raimondii bHLH14* (XP_012490160.1), *Ricinus communis MYC2* (XP_002529965.1), *Manihot esculenta* (OAY62101.1), *Jatropha curcas MYC2* (XP_012083125.1), *Vitis vinifera MYC2* (XP_002266775.1), *Pyrus* x *bretschneideri MYC2* (XP _009375455.1), *Prunus persica* (XP_007219048.1), *Malus domestica MYC2* (XP_008370350.1), and *Citrus sinensis* (KDO86574.1)

**Fig. 2:**
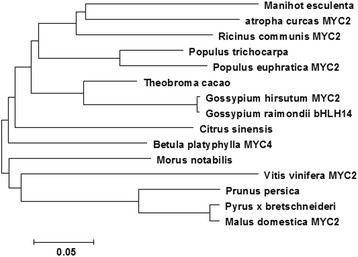
Neighbor-joining phylogenetic tree of *BpMYC4* from *Betula platyphyll* and other *bHLH*s constructed using MEGA 6.0 software. The scale bar represents 0.05 amino acid substitutions per site

The conserved domain of the *BpMYC4*-encoded protein was predicted using the Conserved Domains function in BLAST. The results indicated that the protein contained a bHLH-MYC-N-specific hit within a.a. 25–210, corresponding to the N-terminal region of a family of MYB and MYC transcription factors. In several plant species, members of this family regulate phenylpropanoid biosynthesis. The HLH domains, which bind DNA, were located further downstream at a.a. 312–362, 316–364, and 313–359 (Fig.1). The physicochemical properties of the *BpMYC4* amino acid sequences were analyzed using the Protparam bioinformatics tool of the ExPASy website; the results indicated a molecular mass of 54.41 kDA and an isoelectric point of 5.78. The *BpMYC4*-deduced protein was found to be unstable and hydrophilic. The amino acid sequence of *BpMYC4* was approximately 70% homologous with the bHLHs of *Theobroma cacao*, *Populus trichocarpa*, and *Populus euphratica*. The multiple alignment of the *BpMYC4*-encoded amino acid sequence with other bHLH proteins is shown in Fig.1 (a, b). The neighbor-joining phylogenetic tree, which was constructed using MEGA 6.0 software (Fig. 2), indicates a strong correlation between the *BpMYC4*-encoded amino acids and the amino acids of *Citrus sinensi* (KDO86574.1). The secondary structure of the *BpMYC4* protein revealed using GOR4 software suggested that *BpMYC4* consists of α-helices (37.74%), extended strands (17.39%), and random coils (45.13%).

**Fig. 3:**
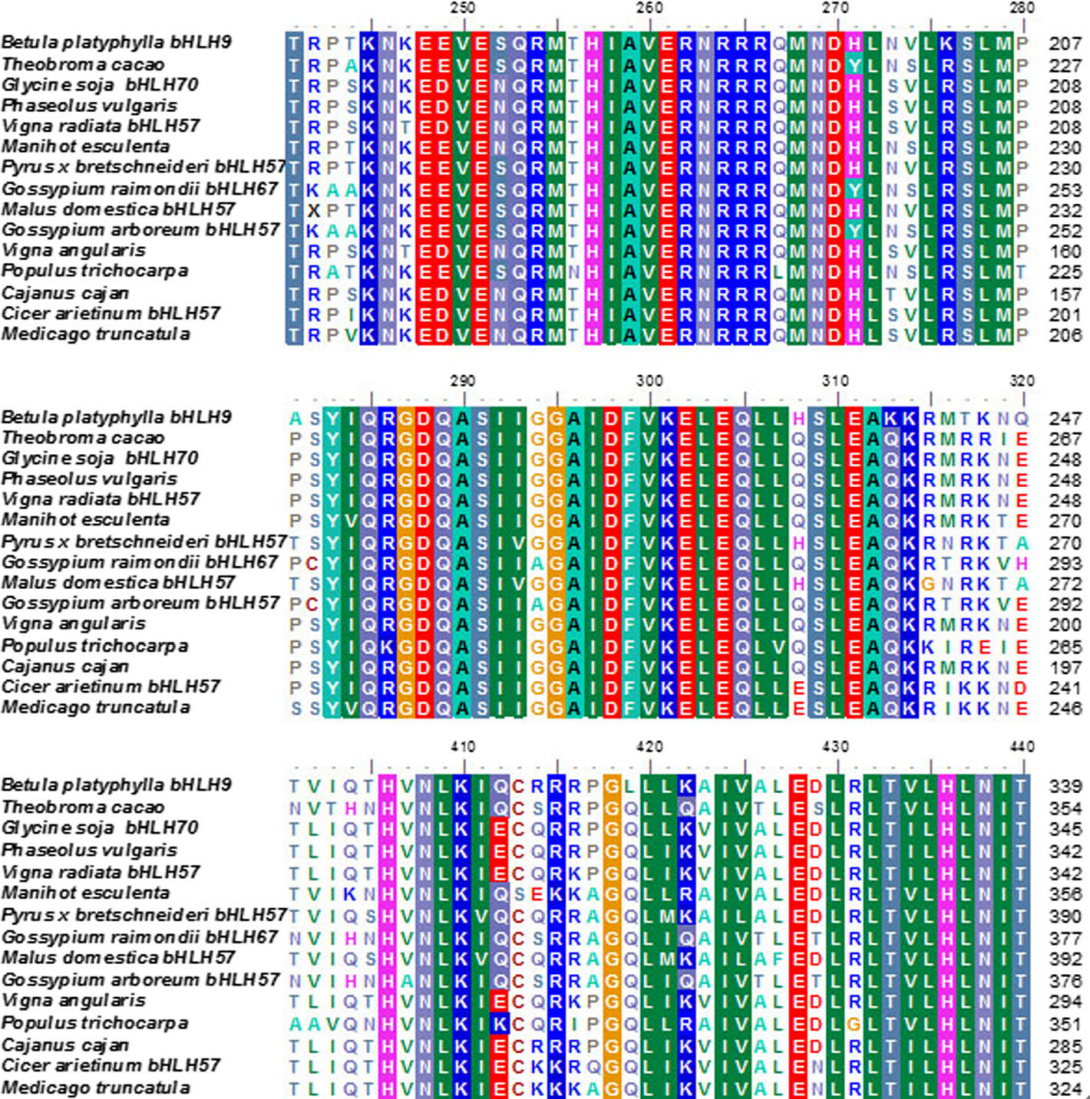
Alignment of the amino acid sequence encoded by *BpbHLH9* with those encoded by the *bHLH*s of other organisms obtained using the ClustalW multiple alignment tool. The aligned sequences were derived from *Manihot esculenta* (OAY48927.1), *Theobroma cacao* (XP_007051418.1), *Glycine soja bHLH70* (KHN06372.1), *Phaseolus vulgaris* (XP_007135328.1), *Vigna radiata bHLH57* (XP_014521636.1), *Pyrus* x *bretschneideri bHLH57* (XP_0 09366590.1), *Gossypium raimondii bHLH67* (XP_012490186.1), *Malus domestica bHLH57* (XP_008376237.1), *Gossypium arboretum bHLH57* (KHF97772.1), *Vigna angularis* (KOM57214.1), *Populus trichocarpa* (XP_006 375,391.1), *Cajanus cajan* (KYP38825.1), *Cicer arietinum bHLH57* (XP_004514916.2), and *Medicago truncatula* (XP_013444538.1)

**Fig. 4:**
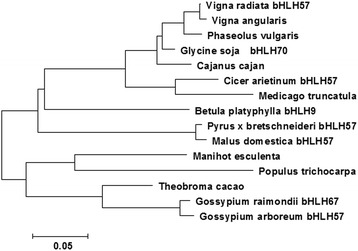
Neighbor-joining phylogenetic tree of *BpbHLH9* from *Betula platyphylla* and other *bHLH*s constructed using the MEGA 6.0 software program. The scale bar represents 0.05 amino acid substitutions per site

The conserved domain of the *BpbHLH9*-deduced protein was predicted using the Conserved Domains function in BLAST. The results indicate that the protein contains a HLH domain at a.a. 180–231, 178–235, and 186–236, with E-box and N-box elements in the vicinity of the HLH domain. The HLH domain is found in specific DNA-binding proteins that act as transcription factors. The physicochemical properties of the *BpbHLH9* amino acid sequences were analyzed using the Protparam bioinformatics tool of the ExPASy website, revealing a molecular mass of 42.90 kDA and an isoelectric point of 6.10. The *BpbHLH9*-encoded protein was found to be unstable and hydrophilic. The amino acid sequence of *BpbHLH9* was approximately 60% homologous with the bHLHs of *Theobroma cacao*, *Glycine soja*, and *Phaseolus vulgaris*. The multiple alignment of the amino acid sequence encoded by *BpbHLH9* with other bHLH proteins is shown in Fig. 3. The neighbor-joining phylogenetic tree constructed using MEGA 6.0 software (Fig. 4) indicated good correlation between the *BpbHLH9*-encoded amino acids with bHLH57 from *Pyrus* x *bretschneideri* and *Malus domestica.*The secondary structure of the *BpbHLH9* protein determined using the GOR4 program indicated that *BpbHLH9* consists of α-helixes (42.74%), extended strands (12.40%), and random coils (44.85%).

### Isolation and analysis of *BpMYC4* and *BpbHLH9* promoter sequences

We obtained *BpMYC4* and *BpbHLH9* gene promoters from *B. platyphylla* using a genome walking approach. The length of the *BpMYC4* gene promoter was 900 bp, and the 5′-untranslated region (5′UTR) was located at the +458-bp site of the promoter. Similarly, the −444-bp site of the *BpbHLH9* gene promoter sequence (length = 1064 bp) also contained a 5′UTR sequence (Additional file 4: Figures S3 and S4). The two promoter sequences were analyzed using PlantCARE software (Tables 1 and 2). The results indicated that the promoter regions of *BpMYC4* and *BpbHLH9* contained multiple eukaryotic cis-acting elements, including TATA and CAAT boxes. In the *BpMYC4* promoter sequence, one abscisic acid response element (ABRE) was found at the +773-bp site, three HSE response elements were located at −487, −677, and −676 bp, one drought response element (MBS) was located at −599 bp, two TC-rich repeating elements involved in defense and stress response were located at +156 and −886 bp, one endosperm expression element (GCN4) was found at −321 bp, and one cis-acting element associated with circadian rhythm was located at −432 bp.

**Table 1:** Putative cis-acting regulatory elements identified in the promoter sequence of *BpMYC4* using the PlantCARE database

Cis element	Position	Sequence	Function of site
5’UTR Py-rich stretch	+458	TTTCTTCTCT	cis-acting element conferring high transcription levels
ABRE	+773	CGCACGTGTC	cis-acting element involved in abscisic acid response
ATGCAAAT motif	−540	ATACAAAT	cis-acting regulatory element associated with the TGAGTCA motif
CAAT-box	−65, −54, +265, +829, +137, +669, −409, −831, −66, −555, +287, −830, +219, +828, +538	CAATT, CAAAT, CAAT, CCAAT	common cis-acting element in promoter and enhancer regions
G-Box	−376, −775, −376, +843, −775	CACGTT, CACGTC	cis-acting regulatory element involved in light response
GCN4_motif	−321	TGTGTCA	cis-regulatory element involved in endosperm expression
HSE	−478, −677, −676	AGAAAATTCG, AAAAAATTTC	cis-acting element involved in heat stress response
MBS	−599	TAACTG	MYB binding site involved in drought-inducibility
Sp1	+49, −642, −629, −661	CC(G/A)CCC, GGGCGG	light-responsive element
TATA-box	−35, +37, +39, +40, +41, +43, +157, −210, −211, −212, −213, +214, −223, +238, −241, +251, −252, +253, +278, +296, −299, −346, +348, +349, +350, +351, −503, −532, −541, −543, −545, +655, +762, +793, +798, −801	TATATAAATC, TCTATATATT, TATATATA, ATATAT, TATA,TTTTA, TATAAAAT, TATAAAA, TATAAA,TATAA, TATACA	core promoter element located next to −30 of transcription start
TC-rich repeats	+156, −886	ATTTTCTTCA, GTTTTCTTAC	cis-acting element involved in defense and stress response
TGG-motif	−619	GGTTGCCA	part of a light-responsive element
circadian	−432	CAANNNNATC	cis-acting regulatory element involved in circadian control

**Table 2:** Putative cis-acting regulatory elements identified in the promoter of *BpbHLH9* by PlantCARE databases

Cis element	Position	Sequence	Function of site
4 cl-CMA2b	+699	TCTCACCAACC	light-responsive element
5’UTR Py-rich stretch	444, −575, −577, −579, −581, +877, +881, +883, +885, +887, +889, +891, +893, +895, +897, +899, +901, +903, +905	TTTCTTCTCT, TTTCTCTCTCTCTC	cis-acting element conferring high transcription levels
ABRE	+146	CACGTG	cis-acting element involved in abscisic acid response
ACE	+147	ACGTGGA	cis-acting element involved in light response
ARE	−317, −665	TGGTTT	cis-acting regulatory element essential for anaerobic induction
ATGCAAAT motif	−352	ATACAAAT	cis-acting regulatory element associated with the TGAGTCA motif
Box I	+231	TTTCAAA	light-responsive element
CAAT-box	+122, +316, −267, −1039, −188, +644, +331, −1044, −187, +643, +275, +1043, +206, −750, −352, −1045	CAAT, CAATT, CAAAT,	common cis-acting element in promoter and enhancer regions
CCAAT-box	−842	CAACGG	MYBHv1 binding site
G-Box	+146, −389	CACGTG, CACATGG	cis-acting regulatory element involved in light response
GAG-motif	−58, −879, −707	AGAGAGT	part of a light-responsive element
GARE-motif	−791	AAACAGA	gibberellin-responsive element
GATA-motif	+19	GATAGGG	part of a light-responsive element
GCN4_motif	−141, −516, −490	TGTGTCA	element involved in endosperm expression
HSE	+262	AGAAAATTCG	cis-acting element involved in heat stress response
I-box	+19	GATAGGG	part of a light-responsive element
L-box	+699	TCTCACCAACC	part of a light-responsive element
LTR	+102	CCGAAA	cis-acting element involved in low-temperature response
MBS	+605	CAACTG	MYB binding site involved in drought-inducibility
Skn-1_motif	−140, −489, +191	GTCAT	cis-acting regulatory element required for endosperm expression
Sp1	+763	CC(G/A)CCC	light-responsive element
GCN4_motif	−141, −516, −490	TGTGTCA	element involved in endosperm expression
TATA-box	−80, −181, +239, +344, −439, −571, −724, −732, −733, −734, −735, −830, +852, +944, −946, −947, −956, −964, −990, −1058	CCTATAAATT, TTTTA, TATA, ATATAT,TAATA	core promoter element around of transcription start
TC-rich repeats	+397, +814	ATTTTCTTCA	cis-acting element involved in defense and stress response
TCA-element	+128, −785	CCATCTTTTT, CAGAAAAGGA	cis-acting element involved in salicylic acid response
TCT-motif	+775	TCTTAC	part of a light-responsive element

*BpbHLH9* also contained plant hormone-response elements, heat-response elements, drought-response elements, defense- and stress-response elements, and endosperm-expression elements, including ABRE (involved in abscisic acid response), the GARE motif (involved in GA response), and TCA [involved in salicylic acid (SA) response], which were located at +146, −791, −785 and +128, respectively. In addition, the *BpbHLH9* promoter contained multiple anaerobic and cryogenic regulatory elements. The *BpMYC4* and *BpbHLH9* promoters were predicted to contain MBS elements that play an important role in regulating the synthesis of secondary metabolites. The presence of these regulatory elements indicated that the cloned sequence conformed to the basic characteristics of the eukaryotic gene promoter, and that the expressions of the *BpMYC4* and *BpbHLH9* genes of *B. platyphylla* were regulated by these elements and responded to a variety of plant hormones and adversities stress.

### Expression patterns of *BpMYC4* and *BpbHLH9* under phytohormone treatments

**Fig. 5:**
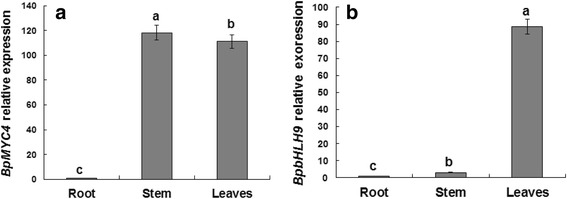
Tissue-specific expressions of *BpMYC4* (**a**) and *BpbHLH9* (**b**) in birch seedlings. The relative expressions of *BpMYC4* and *BpbHLH9* were quantified by quantitative RT-PCR. Reported values are means of three replicates, and the error bars were obtained from multiple replicates. The letters in Fig. 5 indicate a significant difference at the 0.05 level

**Fig. 6:**
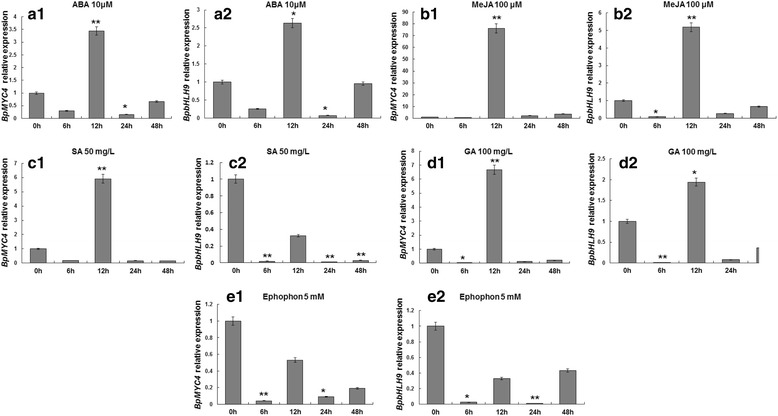
Quantitative assay results for *BpMYC4* and *BpbHLH9* in birch saplings treated by different hormones. The relative expressions of *BpMYC4* and *BpbHLH9* were quantified by quantitative RT-PCR. Reported values are the means of three replicates, and standard errors are indicated as vertical lines on the top of each bar. * Indicates a significant difference between the control and experimental treatments (*P* < 0.05). ** Indicates a highly significant difference between the control and experimental treatments (*P* < 0.01)

RT-PCR experiments were conducted to study the *BpMYC4* and *BpbHLH9* expression patterns in different tissues of *B. platyphylla*. The results showed that the expression of *BpMYC4* in leaves was similar to the expression in the stem and higher than the expression in roots (Fig. 5a). The expression of *BpbHLH9* was higher than in the root and stem tissues (Fig. 5b). Different expression patterns were obtained by treating *BpMYC4* and *BpbHLH9* with different hormones (Fig. 6). The expression of *BpMYC4* was up-regulated by treatment with abscisic acid (ABA; 10 μM), MeJA (100 M), GA (100 mg/L), or SA (50 mg/L) for 12 h. The greatest change in expression was observed in response to MeJA treatment (Fig. 6a), which resulted in a 76-fold increase in *BpMYC4* transcripts during the 12-h treatment. *BpMYC4* transcripts were also up-regulated 2.3 and 3.6 times compared to the control after 24 and 48 h of induction with MeJA, respectively. In contrast, the expression of *BpMYC4* was down-regulated by treatment with the other three hormones for 24 and 48 h. After 12 h of treatment with MeJA, GA, SA and ABA, *BpMYC4* was up-regulated by 76, 6.7, 5.9 and 5.4 times compared to the control group, respectively (Fig. 6a–d). The expression of *BpbHLH9* was increased 5.2-fold by MeJA treatment for 12 h compared to the control (Fig. 6b). However, the expression patterns obtained after treatment with the other hormones were different from those observed for *BpMYC4*. For *BpbHLH9*, treatment with ABA and GA for 12 h resulted in 2.6- and 1.9-fold decreases in expression compared to the control, respectively. Additionally, the expressions of *BpMYC4* and *BpbHLH9* transcripts were decreased 4% and 1% upon ethephon treatment for 6 and 24 h respectively (Fig. 6e), compared to the control. Interestingly, treatment with SA caused a drastic decrease in *BpbHLH9* expression, which reached the minimum after 24 h; compared to the control group, the expression of *BpbHLH9* was reduced by 99% after 24 h of SA treatment (Fig. 6c). These results suggested that SA negatively regulated the expression of *BpbHLH9*. Furthermore, the expression patterns indicated that the two studied bHLH transcription factors had different functions and regulation mechanisms related to plant growth and metabolism.

### *BpMYC4* and *BpbHLH9* localization in the nucleus and cell membrane

**Fig. 7:**
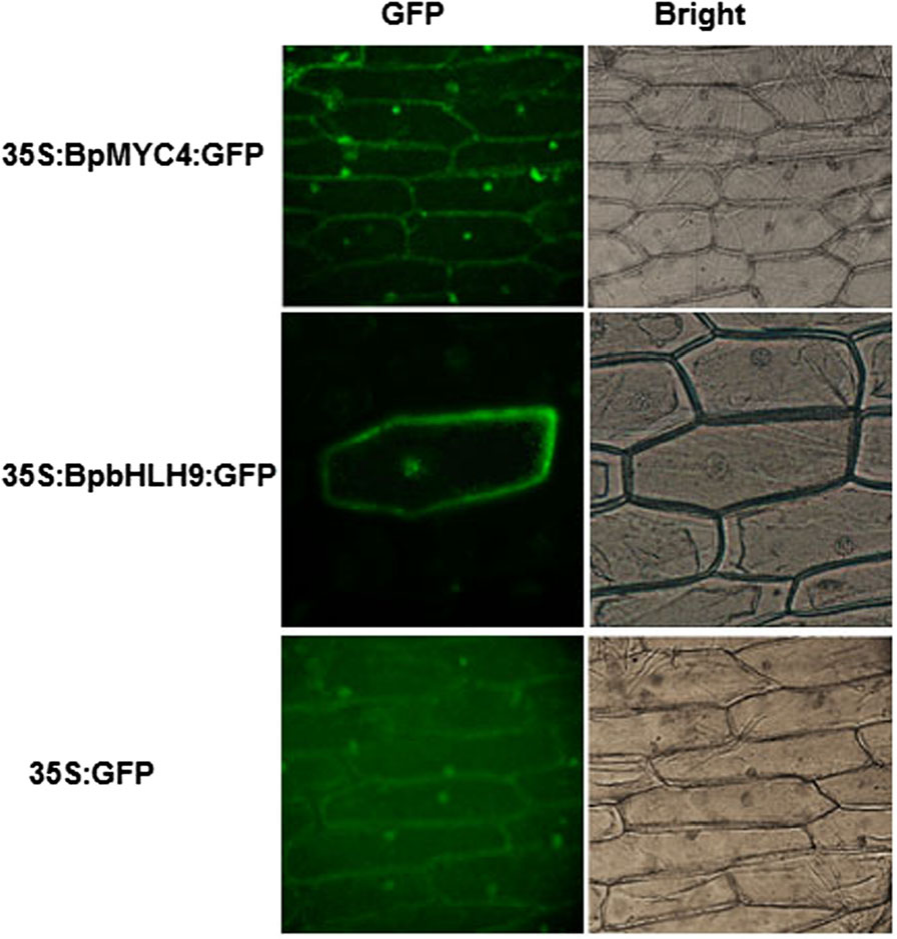
Subcellular localization of BpMYC4/GFP and BpbHLH9/GFP fusion proteins in onion epidermal cells

We investigated the subcellular localization of BpMYC4 and BpbHLH9 in living cells through transient expression assays using onion epidermal cells with constructs that only express GFP, the BpMYC4/GFP fusion, and the BpbHLH9/GFP fusion. Fluorescence microscopy showed that the BpMYC4/GFP and BpbHLH9/GFP fusion proteins were targeted in the nucleus and cell membrane. In contrast, the control (GFP only) distributed throughout the cell (Fig. 7). These findings indicated that BpMYC4 and BpbHLH9 localized in the nucleus and cell membrane.

### Analysis of squalene and total triterpenoid products accumulated in INVScl1 transformants

**Fig. 8:**
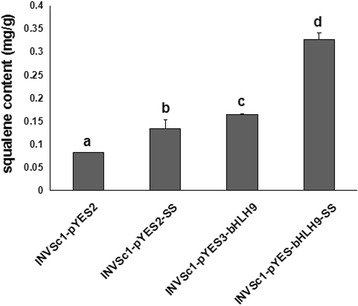
Squalene contents in the cells of the control and transgenic yeasts. The reported values are means of three replicates, and the vertical line on top of each bar indicates standard error. The letters in Fig. 8 indicate a significant difference at the 0.05 level

To study the function of the *BpbHLH9* gene, recombinant yeast and control yeast were induced by galactose for 12 h, and the squalene synthase product was extracted and analyzed by high-performance liquid chromatography (HPLC). We plotted the standard curve of squalene and determined the regression equation to be *y* = 10,000,000*×* – 12,009 (*R* ^2^= 0.9994). After culturing for 12 h, the HPLC-determined squalene content of pYES-BpSS-BpbHLH9 expression yeast cells (0.328 mg/g) was approximately 2.45 times that in pYES2-BpSS expression yeast cells (0.134 mg/g), 1.98 times that in pYES3-BpbHLH9 expression yeast cells (0.165 mg/g), and 3.99 times that in the empty vector control yeast cells (0.082 mg/g, Fig. 8). These results suggested that *BpbHLH9* was involved in triterpenoid synthesis.

**Fig. 9:**
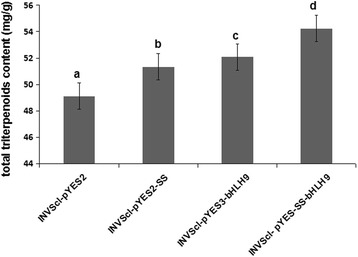
Total triterpenoid contents in the cells of the control and transgenic yeasts. The reported values are the means of three replicates, and the vertical line on top of each bar indicates standard error. The letters in Fig. 9 indicate a significant difference at the 0.05 level

To further study the function of *BpbHLH9*, the recombinant yeast and control yeasts were inoculated for 12 h followed by the extraction of triterpenoids and determination of total triterpenoid content. The standard curve of oleanolic acid was drawn, and the regression equation was determined as *y* = 43.044*×* – 0.6438 (*R* ^2^ = 0.997). The results indicate that when *BpbHLH9* or *BpSS* was transferred into INVScl1 yeast, the content of total triterpenoids in the INVScl1 transformants was significantly increased. When *BpbHLH9* and *BpSS* genes were co-transformed into yeast cells, the total triterpenoid content was further enhanced. The total triterpenoid content in INVScl-pYES-SS-bHLH9 yeast cells was 54.23 mg/g, 10.39% higher than that in the control yeast (INVScl-pYES2). The total triterpenoid content in INVScl-pYES2-SS and INVScl-pYES3-bHLH9 yeasts were increased by 4.49% and 6.00%, respectively, compared to in the control yeast (Fig. 9). These results indicated that *BpbHLH9* gene was involved in the syntheses of squalene and triterpenoids.

### Relative expression of genes related to triterpene synthesis in transgenic birch seedlings

**Fig. 10:**
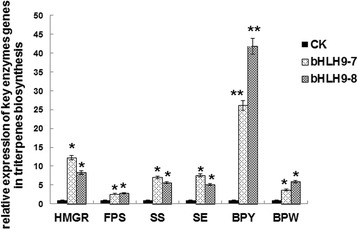
Relative expressions of key enzymes genes (*HMGR*, *FPS*, *SS*, *SE*, *BPY*, *BPW*) related to triterpene synthesis in transgenic birch seedlings. The y-axis values represent multipliers relative to the control. Reported values are the means of three replicates, and standard errors are indicated as vertical lines on the tops of the bars. * Indicates a significant difference between the control and experimental treatments (*P* < 0.05). ** Indicates a highly significant difference between the control and experimental treatments (*P* < 0.01)

To study the role of *BpbHLH9* in triterpenoid synthesis in birch, we obtained *BpbHLH9* transgenic seedlings of Birch*.* (Additional file 5: Figures S5, S6 and S7). The expressions of genes related to triterpene synthesis (HMGR, FPS, SS, SE, BPY and BPW) in the transgenic plants (lines 7 and 8) with high over-expression of BpbHLH9 were detected by real time RT-PCR. The results are shown in Fig. 10. The expressions of *HMGR*, *FPS*, *SS*, *SE*, *BPY* and *BPW* showed different degrees of up-regulation in the bHLH9–7 and bHLH9–8 transgenic plants lines. The greatest change was observed in the expression of *BPY*, which was 26.15 times and 41.76 times that of the wild birch (control), respectively, followed by the expressions of *HMGR*, *SS*, *SE*, *BPW*, and *FPS*.

### HPLC analysis of betulinic acid, oleanolic acid and betulin in *BpbHLH9* transgenic birch

**Fig. 11:**
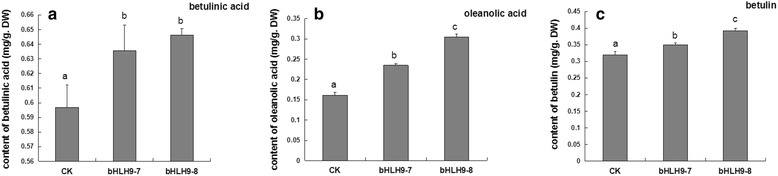
The contents of betulinic acid, oleanolic acid and betulin in *BpbHLH9* transgenic birch. The reported values are the means of three replicates, and the vertical line on top of each bar indicates standard error

To further determine the function of *BpbHLH9* gene in triterpene synthesis, the callus was induced by stem segments of bHLH9–7 and bHLH9–8 lines that greatly overexpress *BpbHLH9*. The triterpenoids were then extracted, and the contents of betulinic acid, oleanolic acid, and betulin in the callus were determined. The standard curves of betulinic acid, oleanolic acid and betulin were drawn, and the respective regression equations were determined as follows: *y*_*1*_ *=* 9 × 10^6^ *x* + 68,020 (*R* ^2^ = 0.9994); *y*_2_ *=* 10^7^ *x* + 43,301 (*R* ^2^ = 0.9993); and *y*_3_ = 10^7^ *x* + 63,763 (*R* ^2^ = 0.9995). The results showed that the contents of betulinic acid, oleanolic acid and betulin in the callus were higher in the bHLH9–7 and bHLH9–8 lines than in the wild type. Compared to the wild type birch (the control), the contents of betulinic acid, oleanolic acid and betulin in bHLH9–7 transgenic callus were increased by 9.49%, 45.35% and 9.56%, respectively. The contents of betulinic acid, oleanolic acid and betulin in bHLH9–8 transgenic birch callus were increased by 11.35%, 88.34% and 23.02% compared to in wild birch, respectively. Among the three tested compounds, the overexpression of BpbHLH9 increased the content of oleanolic acid the most, followed by betulin and betulinic acid (Fig. 11). These results showed that *BpbHLH9* gene was involved in the synthesis of triterpenoids.

## Discussion

Several transcription factors in the bHLH family have been reported to regulate the biosynthesis of different secondary metabolites in various plant species [7–10, 13]. These identified TFs controlled the metabolic flow through the biosynthetic pathway by positively or negatively regulating the expressions of genes that encode key enzymes [18]. TFs in the bHLH family have been shown to play important roles in the biosynthesis of triterpene in plants [5]; for example, TSAR1 and TSAR2 modulated the biosynthesis of triterpene saponin in *Medicago truncatula* [4], and BIS2 was critical for the generation of monoterpenoid indole alkaloid in *Catharanthus roseus* [19]. In this study, we first cloned the full lengths of genes encoding transcription factors MYC4 and bHLH9 in *B. platyphylla* using RT-PCR; these genes were temporarily named *BpMYC4* and *BpbHLH9*, respectively. NCBI BLAST alignment results showed that the amino acid sequence encoded by *BpMYC4* is approximately 70% homologous with the bHLHs of *Theobroma cacao*, *Populus trichocarpa*, and *Populus euphratica*. The conserved domains indicated that the polypeptide sequence encoded by *BpMYC4* contained a bHLH-MYC-N-specific hit. The biosyntheses of phenylpropanoids in tobacco [20], *Arabidopsis* and *Petunia* [21] were shown to be regulated by members of the MYB and MYC families. We hypothesize that *BpMYC4* is involved in secondary metabolism of phenylpropanoids. The amino acid sequence encoded by *BpbHLH9* was approximately 60% homologous with the bHLHs of *Theobroma cacao*, *Glycine soja*, and *Phaseolus vulgaris*. The polypeptide sequence encoded by *BpbHLH9* contained a HLH domain, which is characteristic of DNA-binding proteins that serve as transcription factors. Thus, we think that *BpMYC4* and *BpbHLH9* are two new genes.

We obtained the promoters of *BpMYC4* and *BpbHLH9* from *B. platyphylla* using a genome walking approach; the corresponding fragment lengths were 900 and 1064 bp, respectively. The analyses of the promoter sequences are detailed in Additional file 4: Figures S3 and S4). The *BpMYC4* and *BpbHLH9* promoters contained many RNA polymerase binding sites essential for gene transcription (e.g., TATA and CAAT boxes) along with stress-related cis-elements that allow the *BpMYC4*- and *BpbHLH9*-encoded transcription factors to adapt to the environment. The promoters also contained some light-controlling elements and plant hormone-response elements (e.g., ABRE and the GARE motif), indicating that *BpMYC4* and *BpbHLH9* were regulated by plant hormones. Hiroshi Abe et al. [22] confirmed that AtMYC2, a transcription factor in the bHLH family, was found to act as a transcriptional activator in ABA signaling in *Arabidopsis*. Analyzing the expression patterns of *BpMYC4* and *BpbHLH9* revealed that their expressions were significantly increased after 12 h of treatment with GA (100 mg/L) or ABA (10 μM).

In plants, MeJA and SA were shown to act as signaling molecules of biotic and abiotic stresses [23, 24]; these hormones had either inhibitory or promoting effects that manifested in the form of morphological or physiological changes or defense response [24, 25]. In defense response, plants produce a large quantity of secondary metabolites, including terpenoids. Thus, the biosynthesis of secondary metabolites can be modulated through treatment with these signaling molecules. In a previous study, we demonstrated that MeJA treatment significantly enhanced the contents of total triterpenes, betulinic acid and oleanolic acid in birch and up-regulated the expressions of *FPS, SS, SE, BPY* and *BPW,* which are involved in triterpene biosynthesis [1, 2, 16, 17]. In this study, after 12 h of MeJA treatment, the expressions of both *BpMYC4* and *BpbHLH9* in *B. platyphylla* were significantly up-regulated. These results were similar to the expression patterns of *BpSS* and *BpSE* observed in our previous study [2], indicating that *BpMYC4* and *BpbHLH9* acted as signaling molecules in the biosynthesis of triterpenes in *B. platyphylla*. We hypothesize that MeJA induced *BpMYC4* and *BpbHLH9* accumulation and regulated the downstream expressions of *BpSS* and *BpSE* by binding G-box cis-elements. Our previous study demonstrated that *BpSS* and *BpSE* contained G-box of bHLH regulatory element [2]. Chatel et al. [26] reported that MeJA induced the accumulation of the mRNA that encodes the transcription factor CrMYC1, which is in the bHLH family and specifically binds the G-box element in yeast [27]. Li et al. demonstrated that MYC2 inhibited the activation of genes related to terpene synthase [28]. Clearly, bHLH factors were synthesized de novo in response to MeJA, thereby conferring the MeJA-responsive expression of secondary response genes via binding to G-box-like sequences in their promoters [27]. However, in this study, the expression of *BpbHLH9* decreased after SA treatment. A previous study demonstrated that suppression of the JA pathway by SA functions downstream of the E3 ubiquitin-ligase Skip-Cullin-F-box complex SCF^COI1^, which targeted JASMONATE ZIM-domain transcriptional repressor proteins (JAZs) for proteasome- mediated degradation [29]. Collectively, the above data suggested that JA signaling was inhibited by the SA pathway as a result of the negative regulation of *BpbHLH9* transcriptional factor expression. Ethylene has been reported to enhance anthocyanin, flavonoid, and stilbenoid production in grape (*Vitis vinifera*) cell cultures by upregulating the corresponding biosynthetic genes [30]. Interestingly, in this study, the mRNA expression of *BpMYC4* and *BpbHLH9* decreased after treatment with ethephon, suggesting that *BpMYC4* and *BpbHLH9* acted as negative regulators with specificity for the ethephon signaling pathway. This process might be responsible for the biosynthesis of secondary metabolites.

**Fig. 12:**
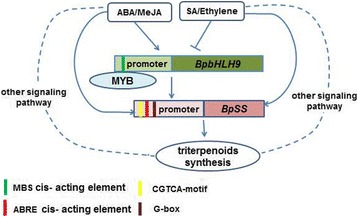
Simplified working model of the role of *BpbHLH9* in hormone-induced triterpenoid synthesis in *Betula platyphylla*. *BpbHLH9* is induced by ABA/MeJA but repressed by ABA/ethylene. BpbHLH9 interacts with MYB to induce *BpSS* expression by binding the G-box cis-elements of the *BpSS* promoter, which subsequently regulates triterpenoid synthesis

TF (transcription factors) have been shown to initiate the expressions of terpenoid synthesis-related genes by binding to the cis-acting elements of the promoters of downstream genes. The transcription factors for terpene biosynthesis specifically bound to the G-Box region to initiate the production of genes related to triterpenoid synthesis [31]. Moreover, the bHLH transcription factors regulated triterpenoid metabolism by initiating *BpSS* and *BpSE* mRNA transcription by binding to the G-Box region [2]. In this study, the accumulation of squalene in INVScl1 transformants was analyzed by HPLC. After 12 h of cultivation, the contents of squalene in different INVScl1 transformants decreased in the following order: pYES-BpSS-BpbHLH9 (0.328 mg/g) > pYES3-BpbHLH9 (0.165 mg/g) > pYES2-BpSS (0.134 mg/g) > empty vector (0.082 mg/g; Fig. 8). The total triterpenoid content in the INVScl-pYES-SS-bHLH9 yeast cells was 10.39% higher than that of the control yeast cells (INVScl-pYES2). The total triterpenoid contents of the INVScl-pYES2-SS and INVScl-pYES3-bHLH9 yeast cells were 4.49% and 6.00% higher, respectively, compared to the control (Fig. 9). The relative expression levels of *HMGR*, *FPS*, *SS*, *SE*, *BPY* and *BPW* were up-regulated significantly in transgenic birch with overexpression *BpbHLH9* (Fig. 10). In addition, the overexpression of *BpbHLH9* enhanced the contents of betulinic acid, oleanolic acid and betulin in birch (Fig. 11). These results confirmed that *BpbHLH9* was involved in the syntheses of squalene and triterpenoids. The main function of MYB transcription factor is to regulate secondary metabolism; it also plays a significant role in plant metabolism and development [32, 33]. In our study, the promoter sequences of *BpMYC4* and *BpbHLH9* were found to have an MBS cis-acting elements that served as an MYB-binding site. We hypothesize that *BpMYC4* and *BpbHLH9* specifically bound MYB transcription factor to initiate the transcription of *BpMYC4* and *BpbHLH9* mRNA or the formation of bHLH/MYB complexes. Schaart et al. [34] reported the control of proanthocyanidin biosynthesis by MYB-bHLH-WD40 regulatory complexes in strawberry [34]. Zhao et al. [35] demonstrated that *MYB-bHLH-WD40* proteins in *Camellia sinensis* controlled multiple enzymatic steps in the biosynthetic pathway of flavonoid production. Therefore, the results of this study suggested that MYB bound to *BpbHLH9* and *BpMYC4* in the MeJA signaling pathway to regulate triterpene synthesis in birch. Based on the results of this study and previous reports [2, 34, 35], we proposed a simplified working model for the role of *BpbHLH9* in hormone-induced triterpenoid synthesis in *B. platyphylla* (Fig. 12). In this model, *BpbHLH9* was induced by ABA/MeJA in response to exogenous hormone signals but repressed by ABA/ethylene. *BpbHLH9* interacted with MYB [34, 35] to initiate *BpSS* expression by binding the G-box cis-acting elements of the *BpSS* promoter [2], which subsequently regulated the synthesis of triterpenoids. The four hormones (ABA, ethylene, ABA and MeJA) mentioned above might have also directly regulated the expression of *BpSS*.

## Conclusions

In this study, we cloned and isolated two genes, *BpMYC4* and *BpbHLH9*, that encoded transcription factors involved in triterpene synthesis. We analyzed the sequences of these genes using bioinformatics software and detected their expression patterns*. BpbHLH9* increased the contents of squalene and total triterpenoids in yeast strains. The recombinant yeast strains lays the foundation for producing 2,3-oxidation squalene and its downstream products by synthetic biology method. Moreover, *BpbHLH9* was involved in triterpenoid synthesis in birch, which could enhance the expression of key genes of triterpenoid pathway and the contents of oleanolic acid, betulinic acid and betulin. The findings provide useful information for future research on the bHLH regulation of terpene synthesis in transgenic birch.

## Methods

### Plant and phytohormone treatment

Different tissues from four-week-old birch seedlings were used in this study. For treatments with GA (100 mg/L), MeJA (100 μM), SA (50 mg/L), ethephon (5 mM), and ABA (10 μM), the root of the plant was completely soaked in WPM liquid medium including the appropriate hormone solution. Birch sapling leaves and stems were harvested at different times after phytohormone treatment.

### Cloning and phylogenetic analysis of genes encoding MYC4 and bHLH9

Complementary DNA and RNA were extracted from birch seedlings using the cetrimonium bromide method reported by Zhang et al. [2]. Using the birch cDNA treated with the above hormones as a template, a pair of degenerate oligonucleotide primers, *MYC4*-F/*MYC4*-R and *bHLH9*-F/*bHLH9*-R (Additional File 1: Table S1), were designed according to the transcriptome data of *B. platyphylla* recorded in our lab. Complete fragments of *BpMYC4* and *BpbHLH9* DNA were recovered, and the DNA was sequenced. Full-length nucleotide sequences of two genes were analyzed using bioinformatics software referring to the methods of Zhang et al. [2].

### Expression analysis of tissue-specific and phytohormone treatment of *BpMYC4* and *BpbHLH9*

The different tissues used to extract RNA were derived from hormone-treated birch. Total RNA was reverse-transcribed into cDNA according to the user’s manual (Takara, China). RT-PCR was carried out according to our previously reported method [2, 16]. The raw data of relative quantification values were calculated according to Livak and Schmittgen with modification [36].

### Isolation of *BpMYC4* and *BpbHLH9* promoters

Genomic DNA of *B. platyphylla* was extracted from fresh tissue. Genome walking products were constructed according to the manufacturer’s protocols (Genome Walking™ Kit, Takara). MYC4-SP1 with bHLH9-SP1, MYC4-SP2 with bHLH9-SP2, and MYC4-SP3 with bHLH9-SP3 (Additional File 1: Table S1) were used as the primers. The promoter sequences were cloned into the vector and subjected to nucleotide sequencing. The putative cis-acting elements of the promoters of *BpMYC4* and *BpbHLH9* were identified by consulting the PlantCARE database.

### Subcellular localization of *BpMYC4* and *BpbHLH9*

The full-length *BpMYC4* and *BpbHLH9* coding regions without termination codons were amplified using MYC4-NcoI-F/R and bHLH9-NcoI-F/R (Additional File 1: Table S1) as primers, respectively. The coding sequences of the two genes were inserted in the NcoI sites of the pCAMBIA1303 vector (Biovector, USA) using an In-Fusion HD Cloning Kit (Takara, China). The fusion constructs pCAMBIA1303-BpMYC4 and pCAMBIA1303-BpbHLH9 and control pCAMBIA1303 were mobilized in strain LBA4404 using the triprental hybridization method. *Agrobacterium*-mediated transient expression in onion epidermal cells has been completed subcellular localization [37]. The infected inner skin of the onion was removed, and images were taken of the BpMYC4/GFP and BpbHLH9/GFP fusion proteins under a fluorescence microscope (Axioskop2plusFL, Zess, Germany).

### Identify of *BpbHLH9* gene in yeast INVScl1 strain

The ORF of the *BpbHLH9* PCR products was amplified using primers bHLH9-BamHI-F and bHLH9- BamHI-R (Additional File 1: Table S1) and inserted into the corresponding BamHI sites of pYES3 (Invitrogen) to construct the fusion plasmid pYES3-BpbHLH9. The plasmid was transferred to the INVScl1 strain (Clontech, USA) and special INVScl1 strains bearing pYES2-BpSS, which were created in our laboratory using the PEG/LiAc method. Single colonies on the corresponding defective medium (SC- Trp and SC-Ura-Trp)were randomly selected and transferred to the corresponding liquid medium to expand the culture. The cells were collected, and the yeast DNA was extracted and BpbHLH9 gene detected by PCR.

### Squalene accumulation in INVScl1 transformants

The transformants were cultured, and the expression of *BpbHLH9* was induced as described in Zhang et al. [2]. The cellular squalene was extracted as reported in Lu et al. [38], and the squalene content was analyzed by HPLC (Waters 1525–2489) [2]. The test was repeated three times.

### Analysis of total triterpenoid content in INVScl1 transformants

The transformants of *BpbHLH9* were induced as described in Zhang et al. [2], and the cells were then collected and dried. Dried yeast cells (50 mg) were weighed and placed in 4 mL of 95% ethanol solution for 24 h. The specific extraction and detection methods for total triterpenoids are found in Ref. [16].

### Determination of betulinic acid, oleanolic acid and betulin in transgenic birch

Dried transgenic birch callus was ground into a powder, and 0.50 g of powder was soaked in 20 mL hydrochloric acid:ethanol solution (2:8 by volume). The solution was refluxed for 3 h in a water bath at 90 °C. After filtration, 15 mL of distilled water was added to 15 mL of the filtrate, and the ethanol was completely distilled off in a water bath at 80 °C. The mixture was extracted three times with 20 mL of ether. The combined extracts were evaporated to dryness at 40 °C and dissolved in 2.5 mL methanol for HPLC analysis.

## Additional files

Additional file 1: Table S1. List of primers used in the study. (DOCX 16 kb)

Additional file 2: Figure S1. Nucleotide sequence and deduced amino acid sequence of *BpMYC4* from *Betula platyphylla*. (DOCX 287 kb)

Additional file 3: Figure S2. Nucleotide sequence and deduced amino acid sequence of *BpbHLH9* from *Betula platyphylla*. (DOCX 193 kb)

Additional file 4: Figures S3 and S4. Promoter sequences of the *BpMYC4* and *BpbHLH9* from birch. (DOCX 137 kb)

Additional file 5: Figures S5, S6 and S7. Identification and acquisition of *BpbHLH9* transgenic seedlings. (DOCX 730 kb)

## Abbreviations

ABA: Abscisic acid
bHLH: Basic helix-loop-helix
FPS: Farnesyl pyrophosphonate synthase
GA: Gibberellin
HMGR: 3-hydroxy-3-methyl glutaryl coenzyme A reductase
HPLC: High-performance liquid chromatography
MeJA: Methyl jasmonate
SA: Salicylic acid
SE: Squalene epoxidase
SS: Squalene synthase
