# Supplementary material for: Cloning and expression of BpMYC4 and BpbHLH9 genes and the role of BpbHLH9 in triterpenoid synthesis in birch
Source: BMC Plant Biol. 2017 Nov 21;17:214. doi: 10.1186/s12870-017-1150-z (PMC5698961; doi:10.1186/s12870-017-1150-z)
Supplement: Supplementary file 1 — List of primers used in the study. (DOCX 16 kb) [file 12870_2017_1150_MOESM1_ESM.docx]

**Table S1 List of primers used in the study**

| No. | Primer name | Direction | Sequence (5′→3′) |
| --- | --- | --- | --- |
| 1 | MYC4-F | Forward | CGAACAAAGCATGGAAGAGCTCATA |
| 2 | MYC4-R | Reverse | CGCAAAGGAAAGTGAAAAAGAAAAA |
| 3 | bHLH9-F | Forward | GGTGCCTCTCTTCTCGTGTT |
| 4 | bHLH9-R | Reverse | GCGGTAGATATGTCTCCTTCTG |
| 5 | MYC4-SP1 | Reverse | TGGTGGGTACTGTTGATGTGGG |
| 6 | MYC4-SP2 | Reverse | CCATTGTCGTCGTTGGCGGATT |
| 7 | MYC4-SP3 | Reverse | TGGACTACGAATTGGAGCCTTT |
| 8 | bHLH9-SP1 | Reverse | CCAGTCTCAAAAGTGGAGGAGAAA |
| 9 | bHLH9-SP2 | Reverse | CAAGCAAAACGCAGGATTAAGGGA |
| 10 | bHLH9-SP3 | Reverse | GGGAGTAACTGGTCCTTGGAGAC |
| 11 | MYC4-NcoI-F | Forward | GGACTCTTGACCATGGGCAGCGAACAAAGCATGGAA |
| 12 | MYC4-NcoI-R | Reverse | GTCAGATCTACCATGGCCTGCTCTAATCTTCTGAGAAGAGCA |
| 13 | bHLH9-NcoI-F | Forward | GGACTCTTGACCATGGCTATGGAATGTTGGTCGCC |
| 14 | bHLH9-NcoI-R | Reverse | GTCAGATCTACCATGGCGCTACCGTTGATGAAGCTTAA |
| 15 | bHLH9-BamHI-F | Forward | TACCGAGCTCGGATCCCTTCTTGGGGGTTACATTTATT |
| 16 | bHLH9-BamHI-R | Reverse | CGTTACTAGTGGATCCTTGGTGCCTCTCTTCTCGTG |
| 17 | TU-F | Forward | TCAACCGCCTTGTCTCTCAGG |
| 18 | TU-R | Reverse | TGGCTCGAATGCACTGTTGG |
| 19 | FPS-F | Forward | CCGCGGGATCTCTGTCATTGA |
| 20 | FPS-R | Reverse | CCAAGGGTGCAGGCAAGAAAT |
| 21 | BPY-F | Forward | CTGCTCAGTTCCTTCAAGTC |
| 22 | BPY-R | Reverse | TTGCCCATGCAGTATGTACC |
| 23 | BPW-F | Forward | TTGAAGACGTGCAAGAACCTG |
| 24 | BPW-R | Reverse | CATCAATGAGGGATAACAAGG |
| 25 | HMGR-F | Forward | GTCATCGGCATCTCCGGTAA |
| 26 | HMGR-R | Reverse | ATGTTACTGGCGTGGGCATT |
| 27 | SE-F | Forward | GCAGACCCTTCACCCATCTTGTTT |
| 28 | SE-R | Reverse | CCACAGTCATTCCTCCCCCAG |
| 28 | SS-F | Forward | CAGAGGTGTAGTGAAAATGAGGCG |
| 30 | SS-R | Reverse | GGTCGTTTGGTAGAGAGATAAGCA |
