# Supplementary figures and images for: Cloning and expression of BpMYC4 and BpbHLH9 genes and the role of BpbHLH9 in triterpenoid synthesis in birch
Source: BMC Plant Biol. 2017 Nov 21;17:214. doi: 10.1186/s12870-017-1150-z (PMC5698961; doi:10.1186/s12870-017-1150-z)

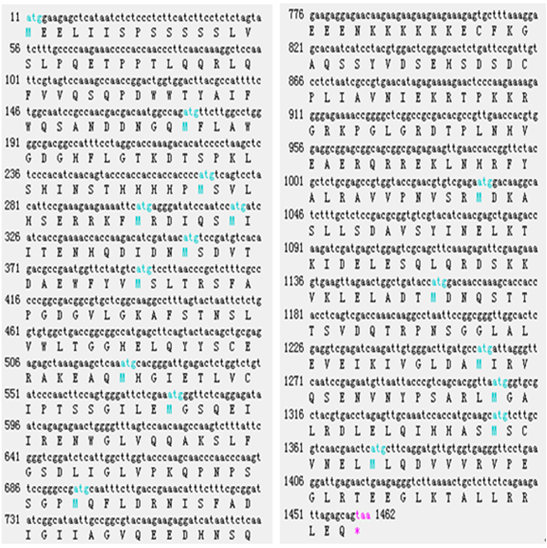


**Fig. S1 Nucleotide and deduced amino acid sequence of *BpMYC4* from *Betula platyphylla***

Supplement: Supplementary file 2 — Nucleotide sequence and deduced amino acid sequence of BpMYC4 from Betula platyphylla. (DOCX 287 kb) [file 12870_2017_1150_MOESM2_ESM.docx]

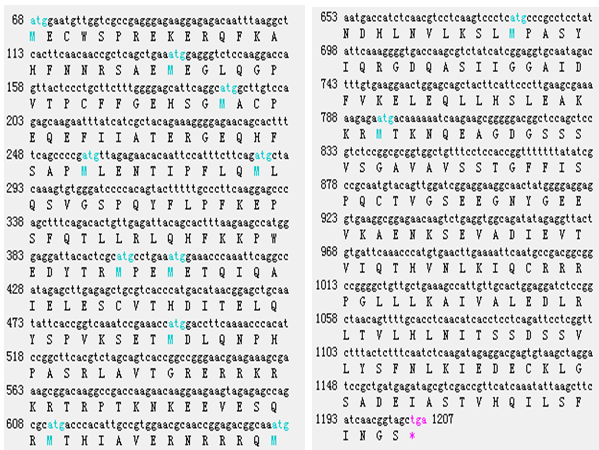


**Fig.** **S2 Nucleotide and deduced amino acid sequence of *BpbHLH9* from *Betula platyphylla***

Supplement: Supplementary file 3 — Nucleotide sequence and deduced amino acid sequence of BpbHLH9 from Betula platyphylla. (DOCX 193 kb) [file 12870_2017_1150_MOESM3_ESM.docx]
