# Supplementary material for: Cloning and expression of BpMYC4 and BpbHLH9 genes and the role of BpbHLH9 in triterpenoid synthesis in birch
Source: BMC Plant Biol. 2017 Nov 21;17:214. doi: 10.1186/s12870-017-1150-z (PMC5698961; doi:10.1186/s12870-017-1150-z)
Supplement: Supplementary file 4 — Promoter sequences of the BpMYC4 and BpbHLH9 from birch. (DOCX 137 kb) [file 12870_2017_1150_MOESM4_ESM.docx]

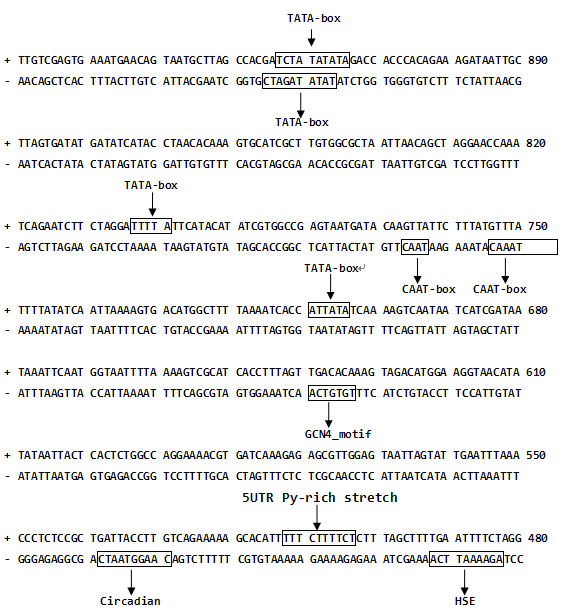

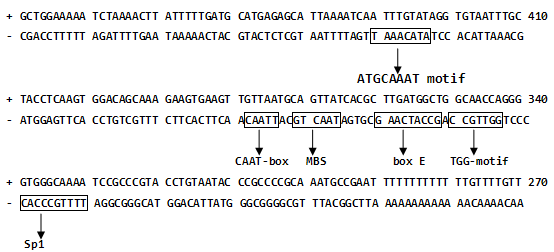

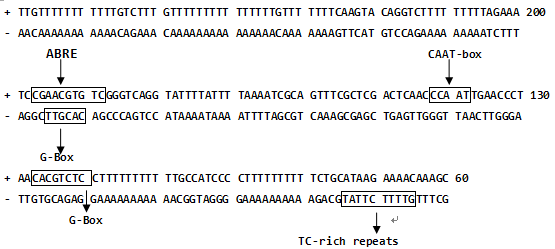


**Fig. S3 The DNA sequence of the *Betula platyphylla BpMYC4* promoter**


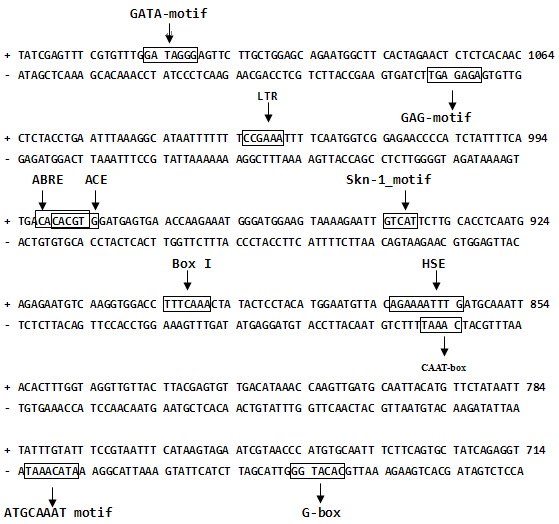

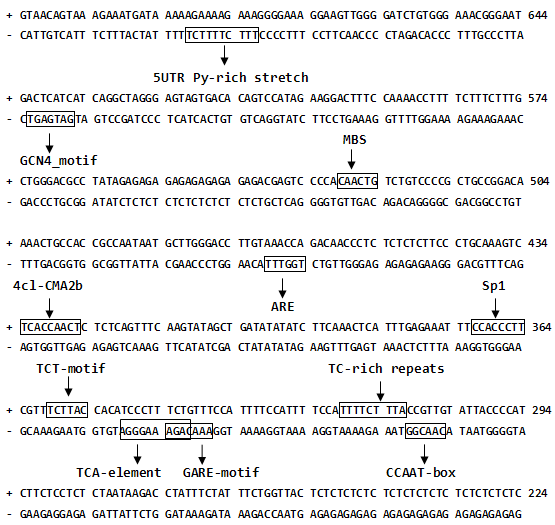

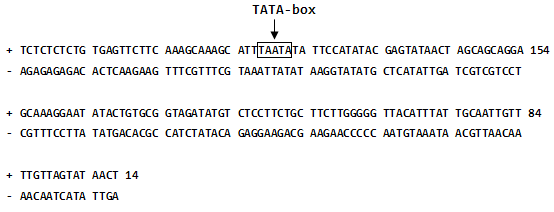


**Fig. S4 The DNA sequence of the *Betula platyphylla BpbHLH9* promoter**
