# Supplementary material for: Cloning and expression of BpMYC4 and BpbHLH9 genes and the role of BpbHLH9 in triterpenoid synthesis in birch
Source: BMC Plant Biol. 2017 Nov 21;17:214. doi: 10.1186/s12870-017-1150-z (PMC5698961; doi:10.1186/s12870-017-1150-z)
Supplement: Supplementary file 5 — Identification and acquisition of BpbHLH9 transgenic seedlings. (DOCX 730 kb) [file 12870_2017_1150_MOESM5_ESM.docx]

The pCAMBIA1303-BpbHLH9 vectors were transferred by *Agrobacterium tumefaciens*-mediated genetic transformation into clones of white birch.Then acpuired 8 transgenic white birch seedlings (bHLH9-1, bHLH9-3, bHLH9-4, bHLH9-7, bHLH9-8, bHLH9-10, bHLH9-11, bHLH9-18) from 22 transformants (bHLH9-1~bHLH9-22) were verified by PCR*.* qRT-PCR analysis showed that the gene expression of *BpbHLH9* were significantly increased in transgenic lines, the overexpression effect were excellent in bHLH9-7 and bHLH9-8 lines.


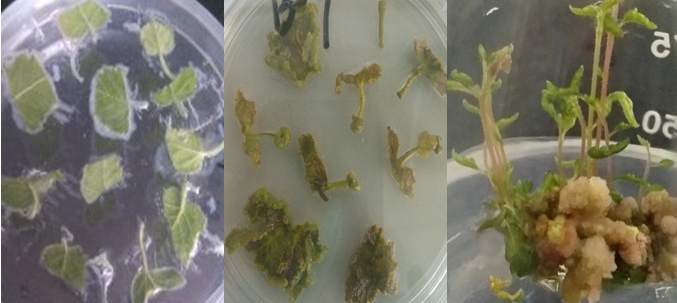

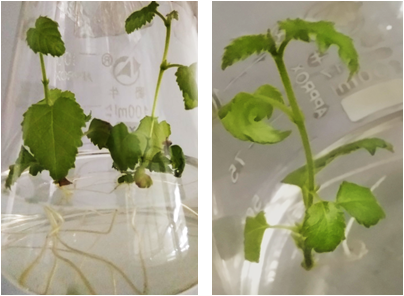


**Fig. S5 *BpbHLH9* transgenic brich seedlings**

**Fig. S6 Identification of transgenic *BpbHLH9* transgenic seedlings by GUS-F/ R primer.**

**M was Marker DL2000, and the last two spots were common birch seedlings and pCAMBIA1303 -BpbHLH9 plasmids. The other spots were the experimental group, named bHLH9-1~bHLH922.**


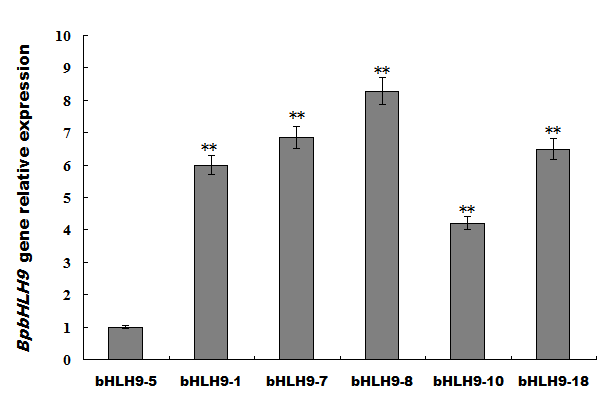


**Fig. S7 The relative expression of *BpbHLH9* gene in transgenic brich**

**(bHLH9-5 : control; bHLH9-1, bHLH9-7, bHLH9-8, bHLH9-10, bHLH9-18: the experimental group)**
